# Inhibiting CXCR2 Remodels the Tumor Microenvironment and Blunts Tumor Progression and Dissemination of High-Grade Serous Carcinoma

**DOI:** 10.1158/2767-9764.CRC-26-0408

**Published:** 2026-09-24

**Authors:** Railey G. Mikeska, Lily Elizabeth R. Feldman, Elizabeth R. Woodruff, Ritsuko Iwanaga, Katharine E. Linder, Tomomi M. Yamamoto, Kayla Sompel, Kimberly R. Jordan, Miriam D. Post, Maia Zoller, Benjamin G. Bitler, Nicole A. Marjon

**Affiliations:** 1 Division of Reproductive Sciences, Department of Obstetrics and Gynecology, University of Colorado Denver, Anschutz Medical Campus, Aurora, Colorado.; 2 Gynecologic Oncology, Department of Obstetrics and Gynecology, University of Colorado Denver, Anschutz Medical Campus, Aurora, Colorado.; 3 Department of Immunology and Microbiology, University of Colorado Anschutz Medical Campus, Aurora, Colorado.; 4 Department of Pathology, University of Colorado Anschutz Medical Campus, Aurora, Colorado.

## Abstract

High-grade serous carcinoma (HGSC) of the ovary, fallopian tube, and peritoneum has an immunosuppressive tumor immune microenvironment (TIME), mediated in part by infiltrating innate immune cells such as macrophages, neutrophils, and myeloid-derived suppressor cells (MDSC). We hypothesize that manipulating these immunoinhibitory cells will improve both response to treatment and outcomes for patients with HGSC. Using publicly available databases, we demonstrate that higher expression of *CXCR2,* a chemotactic receptor for MDSCs and neutrophils, is correlated with decreased overall survival and increased infiltration of neutrophils, monocytes, and M2-like macrophages in HGSC. We targeted the migration of MDSCs and neutrophils to the TIME in the ID8-p53^null^ mouse model of HGSC with the CXCR2-selective inhibitor SB-225002 (CXCR2i) alone or in combination with cisplatin. Tumor weight and dissemination were decreased in mice treated with single-agent cisplatin or CXCR2i. In cisplatin-treated tumors compared with vehicle-treated tumors, there was an increase in suppressive myeloid cells measured by flow cytometry and multispectral immunohistochemistry. Moreover, cisplatin-mediated modulation of tumor immune cell populations was abrogated by the addition of CXCR2i to cisplatin treatment. These findings demonstrate that inhibition of CXCR2 can slow tumor progression and decrease the proportion of immunosuppressive myeloid immune cells in the TIME. Furthermore, these data reveal new insights into detrimental chemotherapy-induced remodeling of the TIME, which should be investigated as mechanistic targets to improve outcomes in HGSC. Overall, our data demonstrate the clinical relevance and therapeutic potential of targeting CXCR2 to prevent the recruitment of tumor-promoting innate immune cells to the TIME.

**Significance:**

Chemotherapy is effective in HGSC but rarely leads to a cure. We demonstrate that chemotherapy induces active CD8^+^ T cells with a concomitant increase in immunosuppressive myeloid cells in the tumor microenvironment. The chemotherapy-induced migration of immunosuppressive myeloid cells is inhibited by a CXCR2i without affecting the activated T cells. We purport that inhibiting CXCR2 with chemotherapy in HGSC will improve T-cell immune surveillance in HGSC and patient outcomes.

## Introduction

The standard treatment strategy for high-grade serous carcinoma (HGSC) of the ovary, fallopian tube, or peritoneum (often referred to as “ovarian cancer”) has not substantially changed in decades (1). The standard of care includes a combination of aggressive cytoreductive surgery and chemotherapy that achieves an 80% remission rate; however, the 5-year survival rate remains low at 48% (1–3). Therefore, novel treatment strategies that bring about durable responses are necessary to extend patient survival and potentially cure this devastating disease.

The tumor microenvironment (TME) of HGSC is complex and plays a role in the development, growth, and treatment response of the malignant cells. One main contributor to the TME is the tumor immune cells, which are primarily immunosuppressive, leading to evasion of immune surveillance. Myeloid cells, including macrophages (4, 5), neutrophils (6, 7), and myeloid-derived suppressor cells (MDSC; refs. 8, 9), are hypothesized to be a major source of TME immunosuppression and treatment resistance. Thus, further investigation is required to understand how to mitigate their activity to improve tumor clearance by the immune system.

Neutrophils are the first responders to infection and injury that initiate a wound healing cascade, including the recruitment of other immune cells and remodeling of the microenvironment (10). In the setting of cancer, a form of chronic inflammation, neutrophils are also recruited to the TME and often promote the growth and metastasis of the primary tumor (11). Furthermore, in HGSC, neutrophils are critical in forming the premetastatic niche in the omentum, allowing tumor spread outside the primary site (7). CXCR1 and CXCR2 (CXCR1/2) are the primary chemotactic receptors present on neutrophils, allowing them to respond to chemokines including CXCL1 (GROα) and CXCL8 (IL8), which are both upregulated in HGSC and correlated with decreased survival (12–16).

Although neutrophils are the primary cell type that expresses CXCR2, MDSCs have also been demonstrated to express CXCR2. MDSCs develop in the setting of chronic inflammation, including cancer, when the maturation of myeloid cells is impeded and immature immune cells that suppress cytotoxic T cells develop. MDSCs are either monocytic (M-MDSC) or granulocytic (PMN-MDSC), which resemble immature monocytes or neutrophils, respectively (17). Preclinical studies in multiple malignancies, including HGSC, demonstrate that increased MDSC tumor infiltration is associated with worse cancer outcomes, including decreased median overall survival (OS) and poorer responses to immunotherapy and standard chemotherapy (6, 8, 18). More specifically, the presence of MDSCs in the TME leads to increased tumor-associated CD8^+^ T-cell exhaustion (19) and increased angiogenesis (8) and promotes tumor immune evasion (6). Therefore, inhibiting the recruitment and expansion of neutrophils and MDSCs to the TME may relieve CD8^+^ T-cell suppression and improve immune surveillance of HGSC tumors.

We hypothesized that inhibiting the migration of myeloid cells, specifically granulocytes and MDSCs, to the TME would decrease tumor growth and metastasis of an immune-intact murine HGSC model. Therefore, in this study, we pharmacologically inhibited the activity of the CXCR2 receptor with a clinically relevant small molecule drug to attenuate the recruitment of MDSCs and neutrophils to the TME.

## Materials and Methods

### Cell culture

The syngeneic (murine) cell line (ID8)-p53^null^ was provided by McNeish and colleagues (20). HGS2-p53^null^, BRCA2^null^, PTEN^null^ (HGS2) cells were provided by Ronald Drapkin, University of Pennsylvania (21). All cells in culture undergo less than 20 passages, are maintained in 5% CO_2_ at 37°C, and undergo monthly mycoplasma testing with the Sigma LookOut Mycoplasma Detection Kit, which was last tested on January 31, 2025 (cat. #MP0035), prior to injection. Cells were authenticated using short tandem repeat profiling through the American Type Culture Collection. ID8-p53^null^ cells were cultured in DMEM (Thermo Fisher Scientific, cat. #10313021) with 4% heat-inactivated FBS (Phoenix Scientific, cat. #PS-100), 1% insulin–transferrin–sodium selenite (ITS; Gibco, cat. #41400-045), 5 μg/mL sodium selenite (Gibco, cat. #41400-045), and 1% penicillin/streptomycin (Thermo Fisher Scientific, cat. #SV30010). HGS2 cells were cultured in media containing DMEM/F-12 (cat. #12634010), 20 mL heat-inactivated FBS, 1% ITS, 5 mL penicillin/streptomycin, 0.05% hydrocortisone (Sigma, cat. #H0888-1G), and 0.005% recombinant human [epidermal growth factor (EGF) protein (R&D Systems, cat. #236-EG-200)]. ID8-p53^null^ conditioned media was generated by culturing ID8-p53^null^ cells at 37°C in 5% CO_2_ until 40% to 60% confluency. The media was then changed and collected 24 to 48 hours later when cells reached 80% to 90% confluency. The collected media was centrifuged and filtered through a 0.22 μm mesh strainer (CELLTREAT, cat. #229747) before being added to sterile ID8 media at a 1:1 ratio.

### *In vivo* HGSC models

All animal experiments were performed in accordance with the Guide for the Care and Use of Laboratory Animals and were approved by the University of Colorado’s Institutional Animal Care and Use Committee (IACUC, protocol #569). All studies utilized 6- to 8-week-old female C57BL/6 immune-intact mice purchased from The Jackson Laboratory (strain #000664). Two independent syngeneic mouse models of HGSC, ID8-p53^null^ and HGS2 cells, were used. ID8-p53^null^ cells were i.p. injected with 1 × 10^6^ cells per mouse 1 week prior to drug treatment, and HGS2 cells were i.p. injected with 1 × 10^7^ cells per mouse 5 weeks prior to drug treatment. There were four experimental groups: (i) vehicle control [daily: 5% DMSO, 30% polyethylene glycol in water intraperitoneally; weekly: phosphate-buffered saline (PBS) intraperitoneally], (ii) cisplatin (weekly, 1 mg/kg intraperitoneally; Selleckchem, cat. #S1166), (iii) CXCR2 inhibitor (CXCR2i; daily, 4 mg/kg intraperitoneally, SB-225002; Selleckchem, cat. #S7651), and (iv) CXCR2i + cisplatin (as above). Mice were treated for 28 days, starting 7 days after cell injection, then euthanized the following day (day 36) from cell injection via CO_2_ inhalation and cervical dislocation.

### Murine bone marrow isolation and MDSC generation

Euthanasia was performed on 7- to 10-week-old female C57BL/6 mice following the IACUC protocol (#569) described above. The femur and tibia bones were collected, and the proximal and distal ends of the bones were removed to allow for the bone marrow to be flushed through the medullary cavity with a 23G × 1″ PrecisionGlide Needle (BD, cat. #305145) and sterile PBS (Thermo Fisher Scientific, cat. #21-040-CV) through a 70 μm mesh strainer (CELLTREAT, 229484) into a conical tube on ice. Isolated murine bone marrow cells were resuspended in the 1:1 ID8 conditioned media, described above, at a concentration of 1 × 10^6^ cells/mL; plated in cell culture dishes; and incubated in a 37°C, 5% CO_2_ incubator for 6 days, with a media change on day 3. On day 6, cells were collected and utilized for experimentation. Alternatively, mice were injected with 1 × 10^6^ ID8-p53^null^ cells (1 × 10^6^ per mouse), and bone marrow from the femur and tibia was isolated on days 35 to 40, and MDSCs were isolated via negative selection using the STEMCELL Murine MDSC Isolation Kit (cat. #19867). MDSC phenotype was confirmed by T-cell inhibition experiments listed below, as well as flow cytometry analysis of samples after 6 days in a 37°C 5% CO_2_ incubator. The phenotypic surface markers used to confirm MDSC phenotypes were anti-CD45 BV 605, anti-Ly6G PE, anti-Ly6C FITC, anti-CD11b APC Cy7, anti-F4/80 BUV 496, and Zombie Violet (Supplementary Table S1).

### Reverse phase protein array

Tumor-bearing mice treated with vehicle or CXCR2i (as described above) were used for bone marrow–derived MDSCs for reverse phase protein array (RPPA) analysis. Mice were treated daily for 28 days, and necropsy of both groups was performed on day 36. The bone marrow from the femur and tibia of the mice was collected following the protocol listed under the heading “Murine bone marrow isolation and MDSC generation,” and MDSCs were isolated using the EasySep Mouse MDSC Isolation Kit (STEMCELL Technologies, cat. #19867). The MDSCs were then washed in PBS and prepared as a dry pellet to be shipped on dry ice to the MD Anderson RPPA core facility (RRID: SCR_017731) per their protocol.

### Phenotypic flow cytometry

Immunophenotypic assessment of murine spleen and omental tissue from tumor-bearing mice (ID8-p53^null^ cells) was performed on day 36 after treatment with cisplatin, CXCR2i, or a combination of cisplatin and CXCR2i as described in the “*In vivo* HGSC models” section. Omental tissue was digested with intermittent mixing for 10 minutes at 5% CO_2_ and 37°C in a solution containing RPMI (Thermo Fisher Scientific, cat. #11875085), collagenase IV (Worthington Biochemical, cat. #LS004188), and DNase I (Thermo Fisher Scientific, cat. #E3101K). Omental tissue was processed by filtering through a 100 μm mesh strainer (EASYstrainer; Greiner Bio-One, cat. #542000) and then a 35 μm mesh strainer (Falcon, cat. #352340) washed twice with PBS. The spleen was processed by pushing it through a 100 μm mesh strainer and then washed twice with PBS. Cells were then incubated with Fc receptor (FcR) blocker (Supplementary Table S1) in FACS buffer (PBS, 1 mmol/L EDTA; Thermo Fisher Scientific, cat. #15575-038) and 2% heat-inactivated FBS for 10 minutes on ice. The cells were washed with FACS buffer and resuspended in Zombie Violet for 20 minutes on ice. Another 20-minute incubation on ice with FACS buffer containing conjugated monoclonal antibodies of interest was performed, and analyses were collected by a BD Acurri C6 Plus (BD) or a NovoCyte Penteon Flow Cytometer (Agilent). Data analyses were executed using FlowJo software (Tree Star). Antibodies were purchased from BioLegend or BD Biosciences; cat. # and RRID can be found in Supplementary Table S1.

### Intracellular cytokine staining

Adherent MDSCs were detached from the plate with a cell scraper, distributed through a 35 μm mesh strainer into a flow cytometry tube (Falcon, cat. #352235), and centrifuged for 5 minutes at 300 *g*. The supernatant was removed, and cells were washed with PBS twice. Cells were then fixed with fixation buffer (BD Biosciences, cat. #554655) for 15 minutes at 4°C and washed with PBS twice. Cells were then permeabilized with Intracellular Staining Permeabilization Wash Buffer (BioLegend, cat. #421002) for 5 to 10 minutes. After fixation and permeabilization, cells were stained with a PE fluorophore-conjugated inducible nitric oxide synthase (iNOS) antibody (Supplementary Table S1), following the manufacturer’s recommended concentration for 20 minutes, on ice and in the dark. Cells were washed again with permeabilization buffer and resuspended in FACS buffer for analysis.

### T-cell suppression assay

Murine bone marrow cells were isolated from healthy C57/BL6 mice and cultured in ID8-p53^null^ conditioned media for 6 days, with the media being changed on day 3. On day 6, cells adhered to the dishes were scraped and resuspended in T-cell media containing RPMI 1640 medium (Thermo Fisher Scientific, cat. #11875085), 10% heat-inactivated FBS, 1% penicillin/streptomycin, 0.1 mmol/L 2-mercaptoethanol, 1% ITS, 1% MEM Non-Essential Amino Acids (Gibco, cat. #11140050), 1% GlutaMax (Gibco, cat. #35050079), and 1% sodium pyruvate (Gibco, cat. #11360070) at varying concentrations relative to the number of T cells. Murine T cells were extracted from the spleen of healthy C57/BL6 mice and negatively selected with the EasySep Mouse T-Cell Isolation Kit (STEMCELL Technologies, cat. #19851). T cells were then resuspended in T-cell media (see above) containing anti-CD28 (BioLegend, cat. #102116) and seeded in an anti-CD3 (BioLegend, cat. #100340) precoated 96-well plate. After 72 hours of MDSC and T-cell coculture, media from each well was collected, and an ELISA MAX Deluxe Set Mouse IFNγ (BioLegend, cat. #430804) was performed. IFNγ concentrations were calculated based on a standard curve.

### Conventional immunohistochemistry

Tumors were paraffin-embedded, sectioned at 5 μm, and mounted on a slide. The slides were deparaffinized prior to antigen retrieval. Antigen retrieval was performed with 10 mmol/L sodium citrate (pH 6.0; CD3 and F4/80) or BORG Tris-based solution (pH 9.5; Biocare Medical, cat. #BD1000; granzyme B) for 10 minutes at 100°C in the NxGen Decloaking Chamber (Biocare Medical). Primary antibodies can be found in Supplementary Table S2; OmniMap anti-rabbit horseradish peroxidase (Roche, cat. #760-4311) was used as the secondary antibody. Detection was performed with ChromoMap 3,3′-diaminobenzidine (Roche, cat. #760-159; RRID AB_2811043). The tissues were then counterstained with Harris hematoxylin and mounted. All three antibody staining protocols were performed on the VENTANA/Roche DISCOVER ULTRA immunostainer. Slides for granzyme B and F4/80 were assessed in a blinded fashion by a gynecologic pathologist and separated into high, intermediate, and low expression based on staining intensity, as well as qualitative percent positive. CitH3 was evaluated by investigators in a blinded fashion and separated into high and low based on staining intensity with extracellular staining present. T cells were quantified blindly by counting CD3^+^ T cells in five fields per slide at 20× magnification and averaged.

### Multispectral immunohistochemistry and image analysis

Multispectral immunohistochemistry (mIHC) analyses were performed using Vectra Automated Quantitative Pathology Systems (Akoya Biosciences) as described previously (22). Tissues were formalin-fixed, paraffin-embedded; sectioned at 4 microns; and placed on slides. Human slides were then stained with antibodies specific for markers CD11b, CD14, CD15, CD33, CD68, cytokeratin (CK), HLA-DR, CD66b, and 4′,6-diamidino-2-phenylindole (DAPI; Supplementary Table S3). Mouse slides were stained with CD11b, Ly6G, Ly6C, MHCII, WT1, CD11c, CD206, F4/80, and DAPI (Supplementary Table S4). All antibody details are provided in Supplementary Tables S2 and S3. All images were deidentified and imaged by the Human Immune Monitoring Shared Resource core (RRID:SCR_021985) on the Akoya Biosciences Vectra Polaris scanner. Regions of interest were selected, and multispectral images were collected with a 20× objective. The inForm software (Akoya Biosciences) was used for tissue and cell segmentation and phenotyping by utilizing a training set of nine representative images to train analysis algorithms. Representative autofluorescence was measured on an unstained control slide and subtracted from study slides. For the human tissue, cell types were defined as follows: CD11b^+^/CD33^+^/HLA-DR^−^ (total MDSC), CD11b^+^/CD33^+^/HLA-DR^−^/CD15^+^ (PMN-MDSC), CD11b^+^/CD33^+^/HLA-DR^−/^CD14^+^ (M-MDSC), CD11b^+^/CD15^+^/CD66b^+^/CD33^−^ (neutrophils), CD14^+^/HLA-DR^+^ (monocytes), CK^+^/HLA-DR^+^ (DR^+^ tumor), and CK^+^/HLA-DR^−^ (DR^−^ tumor). For mouse tissue, cell types were defined as follows: WT1^+^ (tumor epithelial cells), CD11b^+^/MHCII^−^/Ly6C^+^ (M-MDSC), CD11b^+^/MHCII^−^/Ly6G^+^ (PMN-MDSC), F4/80^+^/CD206^+^ (CD206^+^ macrophages), F4/80/CD206^−^ (CD206^−^ macrophages), and CD11c^+^ (dendritic cells).

### MDSC direct coculture/invasion of ID8-p53^null^ spheroids

ID8-p53^null^ cells were plated at 1 × 10^5^ cells per well in DMEM containing 20 ng/mL human EGF (R&D Systems, cat. #236-EG), 20 ng/mL human FGF (R&D Systems, cat. #3718-FB), 1× B27 supplement (Thermo Fisher Scientific, cat. #17504-044), and 0.5% penicillin/streptomycin in a six-well ultralow attachment plate (Corning, Inc., cat. #3471) and cultured for 72 hours in a 37°C, 5% CO_2_ incubator. On day 6 of MDSC culture and day 3 of spheroid culture, MDSCs were stained with CellTracker Green CMFDA (Thermo Fisher Scientific, cat. #C2925) for 30 minutes, then aliquoted at 2.5 × 10^5^ cells per well. Cells were cocultured for 3 days, then fixed with fixation buffer (BD Biosciences, cat. #554655) and stained with a DAPI (Thermo Fisher Scientific, cat. #D1306) nuclear stain. Stained and fixed spheroids were then mounted with SlowFade Glass mounting media (Thermo Fisher Scientific, cat. #S36917) for 24 hours at 4°C, then imaged with a 3i Marianas Inverted Spinning Disk Confocal Microscope using the 20× air objective and laser lines 405 and 488 nm. Quantification of images was performed on SlideBook software (version 6.0.23).

### MDSC invasion assay

Transwell migration plates were coated with Corning Matrigel Growth Factor Reduced (GFR) Basement Membrane Matrix (Corning, cat. #354230) following guidelines for the thick gel method from the Corning website. MDSCs were cultured in ID8-p53^null^ conditioned media for 6 days, then stained with CellTracker Green CMFDA following recommended protocols. MDSCs were then detached from the plate, counted, and resuspended in varying concentrations of CXCR2i in serum-free RPMI 1640, then transferred onto solidified Matrigel. Next, 90 ng/mL of murine GROα (PeproTech, cat. #250-11) was pipetted to the bottom of the Transwell insert, and cells were incubated at 37°C, 5% CO_2_ for 24 hours. After 24 hours, the bottom of the wells was scraped and strained through a cell-strainer cap (cat. #352235) for flow cytometric preparation then collected with the BD Acurri C6 Plus Flow Cytometer (BD).

### 3-(4,5-dimethylthiazol-2-yl)-5-(3-carboxymethoxyphenyl)-2-(4-sulfophenyl)-2H-tetrazolium viability assay

MDSCs were generated using the methods described above in “Murine bone marrow isolation and MDSC generation.” On day 6 of generation, adherent MDSCs were gently detached from the plate with a cell scraper and centrifuged for 5 minutes at 300 *g*. The supernatant was removed, and cells were washed with PBS twice. MDSCs were then plated at 4 × 10^5^ cells per well in RPMI containing 4% heat-inactivated FBS (Phoenix Scientific, cat. #PS-100) and various concentrations of CXCR2i (0–50,000 nmol/L) and cultured for 72 hours in a 37°C, 5% CO_2_ incubator. Similarly, ID8 cells were plated at 1 × 10^5^ at various concentrations of CXCR2i. The CellTiter 96 Aqueous One Solution Reagent (Promega, cat. #G3582) was used to determine cell viability at 72 hours per the manufacturer’s instructions. Briefly, approximately 20 μL of 3-(4,5-dimethylthiazol-2-yl)-5-(3-carboxymethoxyphenyl)-2-(4-sulfophenyl)-2H-tetrazolium (MTS) solution was added to 100 μL of cell culture media at 72 hours, and the mixture was incubated at 37°C for 1 hour. Cell viability was then measured on a plate reader at 570 nm.

### Tissue microarray

A previously constructed tissue microarray (TMA) comprising serous tumors from patients with ovarian cancer treated at the University of Colorado was provided by the Gynecologic Tissue and Fluid Bank [Colorado Multiple Institutional Review Board (COMIRB) #17–7788; refs. 22–24]. The generation of the TMA was retrospective, and patient information was deidentified; thus, written informed consent was not required as deemed by COMIRB, in accordance with the ethical standards defined by the Declaration of Helsinki.

### Statistical analysis

All *in vitro* experiments were performed in triplicate, and the *in vivo* tumor progression was performed in duplicate with 10 mice per group in each experiment. Survival comparison for publicly available data was performed using Kaplan–Meier with log rank (used restrictions: autoselect best cutoff; *CXCR2* as our gene; ovarian cancer, all histologies). For OS and progression-free survival (PFS) for the human TMA, Kaplan–Meier with log rank was used. Pairwise comparisons were performed using independent *t* tests for the analysis of the TMA, as well as for *in vitro* experiments. For both *in vitro* and *in vivo* studies with multiple treatment groups, outliers were removed using the ROUT method set at 1%. Data were then evaluated using an ordinary one-way ANOVA with multiple comparisons. *Post hoc* corrections were performed as needed using the Sidak correction and false discovery rate multicomparison correction (q < 0.05). The mIHC was evaluated by total tumor area, total cell count, and cell densities of positive and negative cells for each phenotype. Significance was set at *P* ≤ 0.05. All statistical analyses were conducted in Prism GraphPad version 9. Error bars are shown as the standard error of the mean.

### Publicly available datasets

Yoshihara and colleagues (25) described a cohort of 110 patients with HGSC. The gene expression microarray data and clinical metadata for patients in this cohort were made available via the Gene Expression Omnibus data repository. Patients with a recorded platinum-free interval (PFI) between 0 and 61 months (*n* = 76) were stratified based on mRNA upregulation (above/below median) of target proteins CXCR1 and CXCR2 as labeled. The log-rank test was used to test the difference in OS between the stratified patient groups. Cox proportional hazard ratios were generated using the coxph command in the Survival package in R (version 4.3.0). The Cancer Genome Atlas Ovarian Serous Cystadenocarcinoma (PanCancer Atlas) was examined for *CXCR2* mRNA expression using the Pan-Cancer Kaplan–Meier Plotter selected for ovarian cancer. OS was analyzed using autoselect best cutoff (26). The data were accessed on August 17, 2023. To determine immune cell infiltration correlated with *CXCR2* mRNA expression, imputation analysis was performed with TIMERv2 and CIBERSORT platforms accessed on December 11, 2024 (27).

## Results

**Figure 1.**
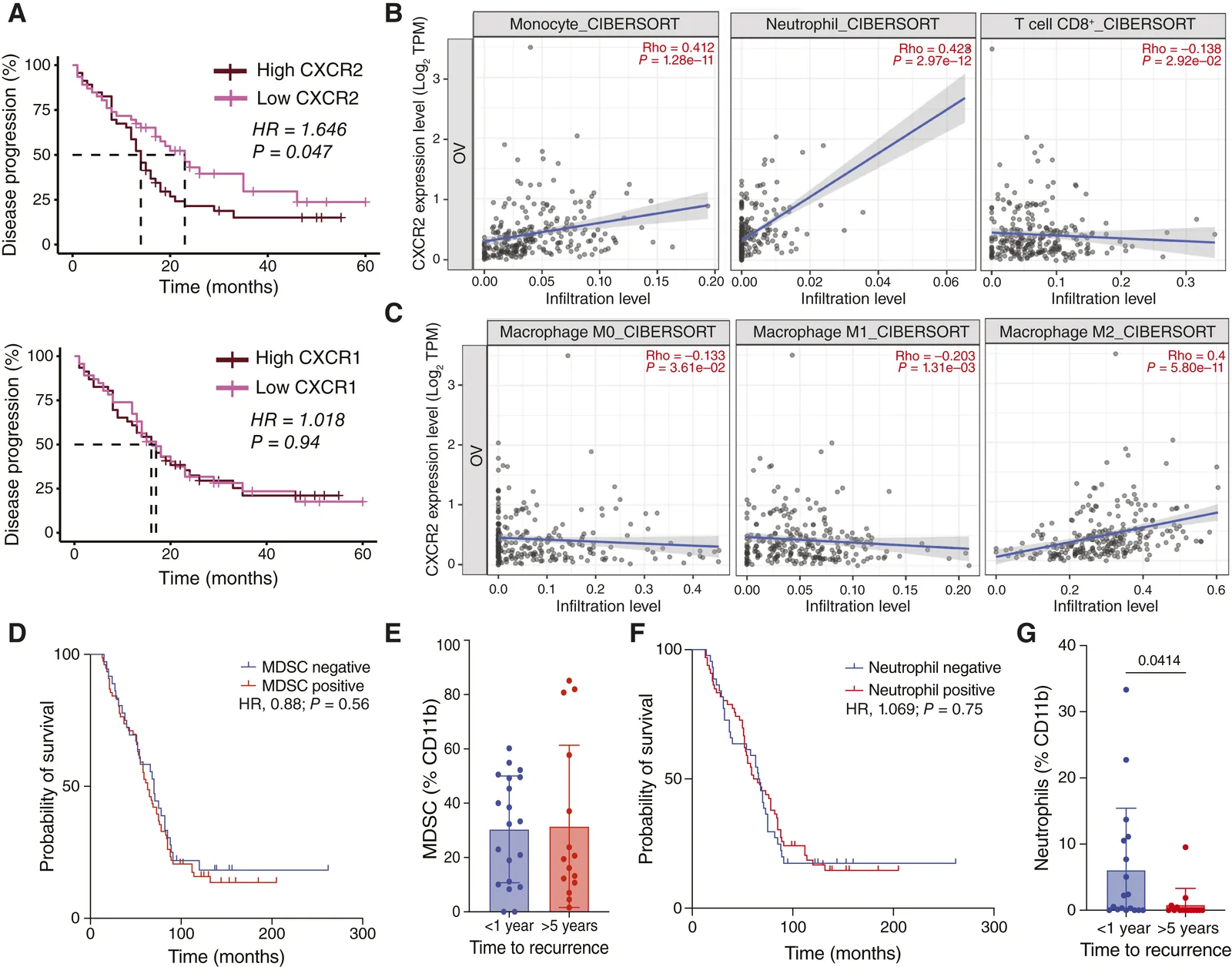
*CXCR2* and immune cell infiltration are correlated with survival in human HGSC. **A,** Probability of PFI vs. time (in months) of patients with HGSC using *CXCR2*^high^ or *CXCR2*^low^ and *CXCR1*^high^ or *CXCR1*^low^ tumors. **B,***CXCR2* gene expression determined by RNA-seq in The Cancer Genome Atlas (TCGA) samples correlated with infiltration levels of monocytes, neutrophils, and CD8^+^ T cells in tumors of patients with ovarian cancer. **C,***CXCR2* gene expression determined by RNA-seq in TCGA ovarian cancer samples correlated with infiltration levels of macrophage subtypes including M0, M1 (antitumor), and M2 (protumor). **D,** Probability of OS vs. time (in months) of HGSC separated by MDSC positive vs. negative tumors and (**E**) time to recurrence (<1 year or >5 years) vs. MDSC levels in patient tumors. **F,** Probability of survival (OS) vs. time (in months) of patients with HGSC using neutrophil positive or negative and (**G**) time to recurrence (<1 year or >5 years) vs. neutrophil levels. Error bars, SD. Statistical tests: Log-rank test, linear regression, and unpaired Student *T* test.

Using publicly available data, we demonstrate that *CXCR2* expression correlates with PFI, OS, and immune cell infiltration in ovarian cancer. CXCR1 and CXCR2 are key chemokine receptors expressed primarily on neutrophils and MDSCs (13, 28, 29). On human neutrophils, CXCR1 is required primarily for degranulation, whereas CXCR2 is integral for migration (30). We explored a previously published dataset of mRNA expression from a cohort of 110 patients with HGSC for correlations between HGSC PFI and *CXCR1 or CXCR2* expression (25). We found a statistically significant correlation between decreased PFI and elevated levels of *CXCR2* (*P* = 0.047; HR, 1.646), but not *CXCR1* (*P* = 0.94; HR, 1.018), indicating a potential role of CXCR2 in driving platinum response in this disease setting (Fig. 1A). Similarly, analysis of publicly available bulk RNA sequencing (RNA-seq) of primary human HGSC demonstrated that *CXCR2* is correlated with decreased OS in human ovarian cancer (52.8 vs. 43.6 months for low vs. high expression, respectively; HR, 1.43; log-rank *P* = 0.011), but *CXCR1* is not (49 vs. 44.7 months for low vs. high expression, respectively; HR, 1.3; log-rank *P* = 0.073; Supplementary Fig. S1A and S1B; KMPlotter August 17, 2023; ref. 26). We next examined a potential underlying effect of elevated CXCR2 expression on survival by evaluating the correlation of *CXCR2* expression with immune cell infiltration in HGSC using the cellular deconvolution platform TIMER v2.0 (27). We focused on the associations between *CXCR2* expression and the infiltration of key immune cell populations. Using the CIBERSORT (31) algorithm, *CXCR2* expression correlated with increased infiltration of monocytes (Rho = 0.412; *P* < 0.001) and neutrophils (Rho = 0.423; *P* < 0.001; Fig. 1B). Additionally, CXCR2 expression is correlated with decreased M0 (Rho = −0.133; *P* = 0.004) and M1 (antitumor) macrophages (Rho = −0.203; *P* = 0.001) and increased M2 (protumor) macrophages (Rho = 0.4; *P* < 0.001; Fig. 1C). No associations were found between *CXCR2* expression and activated dendritic cells (Rho = 0.12; *P* = 0.059), activated NK cells (Rho = −0.113; *P* = 0.075), or B cells (Rho = −0.035; *P* = 0.58). Interestingly, *CXCR2* is correlated with decreased CD8^+^ T cells (Rho = −0.138; *P* = 0.003; Fig. 1B) although it has no association with regulatory T cells (Rho = −0.083; *P* = 0.19). These results highlight the clinical relevance of *CXCR2* expression in HGSC tumors and underscore the correlation between poor prognosis and an immunosuppressive tumor immune microenvironment (TIME).

Given the limitations of publicly available datasets, especially the level of clinical annotation, we performed mIHC on a curated TMA with 135 HGSC tumors (24, 32) to interrogate infiltration levels of myeloid-derived immunosuppressive cells. Clinical annotations included known prognostic factors for outcomes in patients with HGSC (e.g., BRCA mutations, optimal debulking surgery) and survival. MDSCs (CD11b^+^CD33^+^HLA-DR^−^) were detectable in 94.6% of tumors represented in the TMA, comprising 0% to 26% of total cells and up to 78% of CD11b^+^ myeloid-derived cells. PMN-MDSCs (CD11b^+^CD33^+^HLA-DR^−^CD15^+^) ranged from 0% to 22% of total cells, and M-MDSCs (CD11b^+^CD33^+^HLA-DR^−^CD14^+^) ranged from 0% to 19% of total cells. We found no correlation between MDSCs and OS (median OS: 70 vs. 63 months for MDSC low vs. high, respectively; log-rank *P* = 0.58) or PFS (median PFS: 22 vs. 22 months for MDSC low vs. high, respectively; log-rank *P* = 0.98; Fig. 1D; Supplementary Fig. S1C). There was no correlation between CD33^+^ cells and OS (median OS: 64.5 vs. 67.5 months for CD33^+^ low vs. high, respectively; log-rank *P* = 0.80; Supplementary Fig. S1D). Although CD33 is used as a marker of MDSCs and is correlated with OS in other datasets (9), CD33 is not selective for MDSCs and is therefore not a sufficient marker. Neutrophils (CD11b^+^CD15^+^CD66b^+^CD33^−^) were detectable in 58% of tumors and made up a smaller proportion of CD11b^+^ myeloid cells (0%–33%). When evaluated as a discrete variable (i.e., presence vs. absence of neutrophils), the presence of neutrophils did not correlate with OS (median OS: 66.5 vs. 63 for neutrophil negative vs. positive, respectively; log-rank *P* = 0.75) or PFS (median PFS: 25 vs. 21 months for neutrophil negative vs. positive, respectively; log-rank *P* = 0.084; Fig. 1F; Supplementary Fig. S1E).

We then evaluated patients with clinically relevant short time to recurrence (<1 year) compared with long time to recurrence (>5 years). There was no difference in the percentage of MDSCs (30.3% vs. 31.4% for <1 vs. >5 years time to recurrence, respectively; *P* = 0.9; Fig. 1E), but long recurrence was associated with decreased neutrophils compared with short recurrence (6.1% vs. 0.83% for <1 vs. >5 years time to recurrence, respectively; *P* = 0.041; Fig. 1G). There was also no association between MDSCs and the ability to perform an optimal debulking surgery (3.4% vs. 3.3% for suboptimal vs. optimal debulking surgery, respectively; *P* = 0.93; Supplementary Fig. S1F), the need for neoadjuvant chemotherapy (1.9% vs. 3.3% for neoadjuvant vs. upfront debulking, respectively; *P* = 0.25; Supplementary Fig. S1G), or BRCA mutational status (3.9% vs. 3% for BRCA positive vs. negative, respectively; *P* = 0.428; Supplementary Fig. S1H). These characterizations demonstrate that the presence of neutrophils in the TIME strongly correlates with PFS, independently of other common prognostic factors.

**Figure 2.**
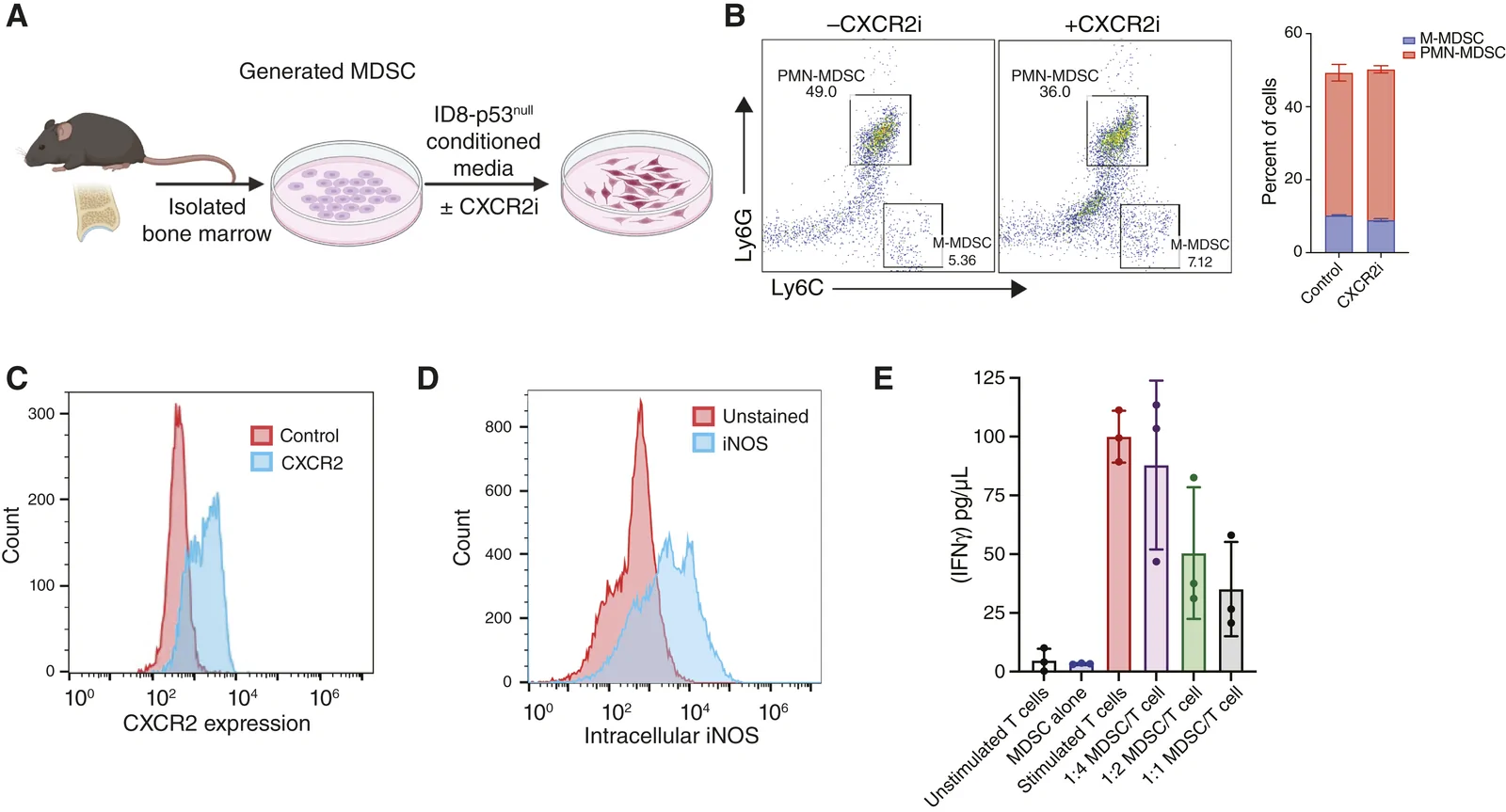
*Ex vivo*–derived MDSCs express phenotypic characteristics and perform immunosuppressive functions. **A,***Ex vivo* murine MDSC generation schema. **B,** Flow cytometric confirmation of immunophenotypic murine MDSC markers in the presence and absence of CXCR2i (100 nmol/L). **C,** CXCR2 expression on generated MDSCs by flow cytometry. **D,** Intracellular iNOS expression in MDSCs. **E,** MDSC-mediated suppression of IFNγ secretion by T cells. Error bars, SD. Statistical test: One-way ANOVA.

Given that CXCR2 is predominantly expressed on granulocytes and that its mRNA expression is correlated with both patient survival and tumor immune cell infiltration, we investigated CXCR2 as a pharmacologic target in a translational mouse model of HGSC. To evaluate the effects of CXCR2 inhibition on MDSCs and tumor-educated neutrophils, we generated MDSCs *ex vivo*. It is to be noted that due to the ambiguity of cell surface markers, we will group PMN-MDSC and tumor-educated neutrophils as PMN-MDSCs. The bone marrow from healthy, non–tumor-bearing mice was isolated, and the cells were cultured in conditioned media from ID8-p53^null^ mouse ovarian cancer cells. The cells were cultured with or without the CXCR2-selective inhibitor (CXCR2i) SB225002 (Fig. 2A). We confirmed the generation of MDSCs by both cell surface markers and inhibitory function. The generated MDSCs were assessed for cell surface markers, including CD11b, Ly6G, and Ly6C, and more than 50% of the cells generated were MDSCs, with PMN-MDSCs being the primary cell type generated, marked by CD11b^+^Ly6G^+^Ly6C^low^ (Fig. 2B). Cell surface expression of CXCR2 was confirmed by flow cytometry on the generated MDSCs (Fig. 2C). The addition of the CXCR2i did not change the proportion of cells that differentiated into MDSCs or into PMN-MDSC versus M-MDSC, suggesting that MDSC differentiation is independent of CXCR2 signaling in this context (Fig. 2B).

To further demonstrate that our generated MDSCs were phenotypically consistent with MDSCs, we confirmed that the cells expressed iNOS (Fig. 2D), as previously described (33). iNOS-mediated nitric oxide is a secreted factor responsible for T-cell inhibition (33). To confirm this inhibitory function in the MDSCs, a T-cell suppression assay was performed with CD3/CD28-activated T cells that were cultured in various ratios with generated MDSCs and evaluated for secretion of IFNγ. Media from unstimulated T cells contained an average of 49 pg/mL of IFNγ, compared with an average of 1,075 pg/mL in media from T cells stimulated with CD3/CD28. Stimulated T cells were then cocultured with increasing MDSCs, with a 1:4 MDSC/T-cell ratio being the lowest number of MDSCs, and IFNγ levels were measured. Notably, MDSCs alone produced insignificant levels of IFNγ (Fig. 2E). The data from the independent experiments were normalized to the stimulated T-cell control. There was a dose-dependent decrease in IFNγ with increasing MDSC:T-cell ratio. At a 1:4 MDSC/T-cell ratio, there was no inhibition of IFNγ secretion, but at a 1:2 MDSC/T-cell ratio, there was a 50% decrease (*P* = 0.049), and at a 1:1 MDSC/T-cell ratio, there was a 65% decrease (*P* = 0.01) in IFNγ secretion (Fig. 2E). These results provide further evidence that our *ex vivo* generated MDSCs function as T cell–suppressive cells.

**Figure 3.**
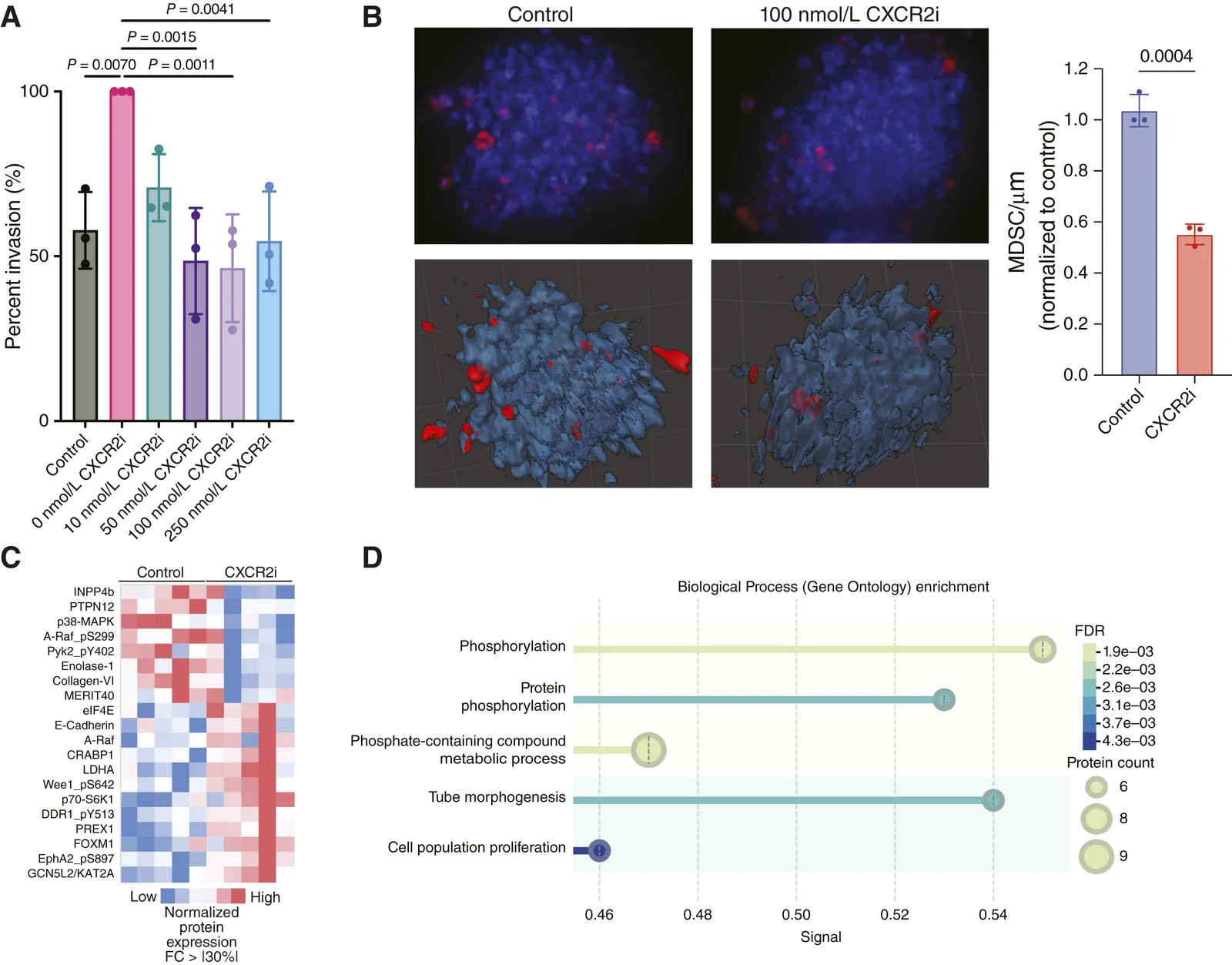
CXCR2-mediated migration of MDSCs is inhibited by CXCR2i. **A,** Transwell invasion assay to chemoattractant GROα with varying CXCR2i concentrations (0–250 nmol/L). **B,** Confocal spinning disk analysis of the invasion characteristics of MDSCs to ID8-p53^−/−^ spheroids in the presence of CXCR2i (100 nmol/L; nuclei – DAPI, blue; MDSCs, red). **C,***In vivo* effect of CXCR2i on protein expression by MDSCs via RPPA analysis. **D,** Gene enrichment analysis based on RPPA analysis. FDR, false discovery rate. Error bars, SD. Statistical tests: (**A**) One-way ANOVA with Tukey multicomparison test and (**B**) unpaired *t* test.

In addition to their function as T-cell suppressors, MDSCs also actively respond to signals secreted by tumor cells and other immune cells, such as IL8 and GROα, two chemokines that attract MDSCs by binding to the CXCR2 receptors on the MDSC surface (13, 29). Migration and invasion via CXCR2, therefore, represent a targetable approach to attenuate immunosuppression in HGSC tumors. We analyzed the effects of CXCR2i on the invasion of *ex vivo* generated MDSCs through Matrigel (Fig. 3A) and into ID8-p53^null^ (mouse model of HGSC) tumor spheroids (Fig. 3B) by Transwell invasion assay. Fluorescently labeled MDSCs were seeded into the top well and allowed to invade through Matrigel toward GROα. MDSC invasion was evaluated in the presence of increasing concentrations of CXCR2i. At 50 to 100 nmol/L CXCR2i, there was approximately 50% inhibition of MDSC invasion (*P* = 0.002 and 0.001, respectively), demonstrating that CXCR2i in MDSCs functions to inhibit migration (Fig. 3A). To ensure that there were not fewer cells due to decreased viability of MDSCs, we performed a viability assay with MDSCs in the presence of increasing concentrations of CXCR2i. There was no observed change in the viability of the MDSCs (Supplementary Fig. S2A).

Next, nonadherent ID8-p53^null^ tumor spheroids were formed and cocultured with stained MDSCs. The tumor spheroids and MDSCs were cultured in the presence or absence of CXCR2i. MDSC spheroid infiltration was monitored via confocal imaging. CXCR2i decreased spheroid-associated MDSCs by 53% (*P* = 0.0004; Fig. 3B), suggesting that ID8-p53^null^ cells effectively attract MDSCs in a CXCR2-dependent fashion. Together, CXCR2 inhibition effectively reduced MDSC migration and spheroid invasion.

To determine the mechanism of CXCR2 targeting, we evaluated signaling in MDSCs isolated from CXCR2i-treated tumor-bearing mice. We employed a small-scale functional proteomics assay, RPPA (34), on MDSCs isolated from the bone marrow of tumor-bearing mice treated or untreated with CXCR2i for 28 days (35). RPPA provides the expression and phosphorylation levels of 432 proteins, and from this, we identified 20 candidate proteins with statistically significant differences in expression based on CXCR2i treatment (*P* value < 0.05, unpaired *t* test) and a log_2_ fold change ≥30% (Fig. 3C). Gene enrichment analysis was performed, and the phosphorylation pathways were those noted to be most affected by CXCR2i, which is consistent with the downstream signaling from CXCR2 (Fig. 3D; ref. 36). P38 (gene: *MAPK14*) mitogen-activated protein kinase (p38-MAPK), a signaling protein downstream of CXCR2, was downregulated in the CXCR2i-treated mice, validating the on-target activity of the CXCR2i. Additionally, Enolase 1 (ENO1), an important enzyme in the migration and invasion of neutrophils and other immune cells (37, 38), was downregulated in the MDSCs from CXCR2i-treated mice compared with control MDSCs (Fig. 3C). However, those proteins that were upregulated, FOXM1 and EPHA2, may also contribute to immune cell tumor infiltration (Fig. 3C; refs. 38–40). These candidate proteins are targets for future studies, as some have not yet been described in immune cells. Based on our findings, the inhibition of CXCR2 attenuates MDSC invasion and downregulates invasion-promoting factors, including ENO1, in HGSC.

**Figure 4.**
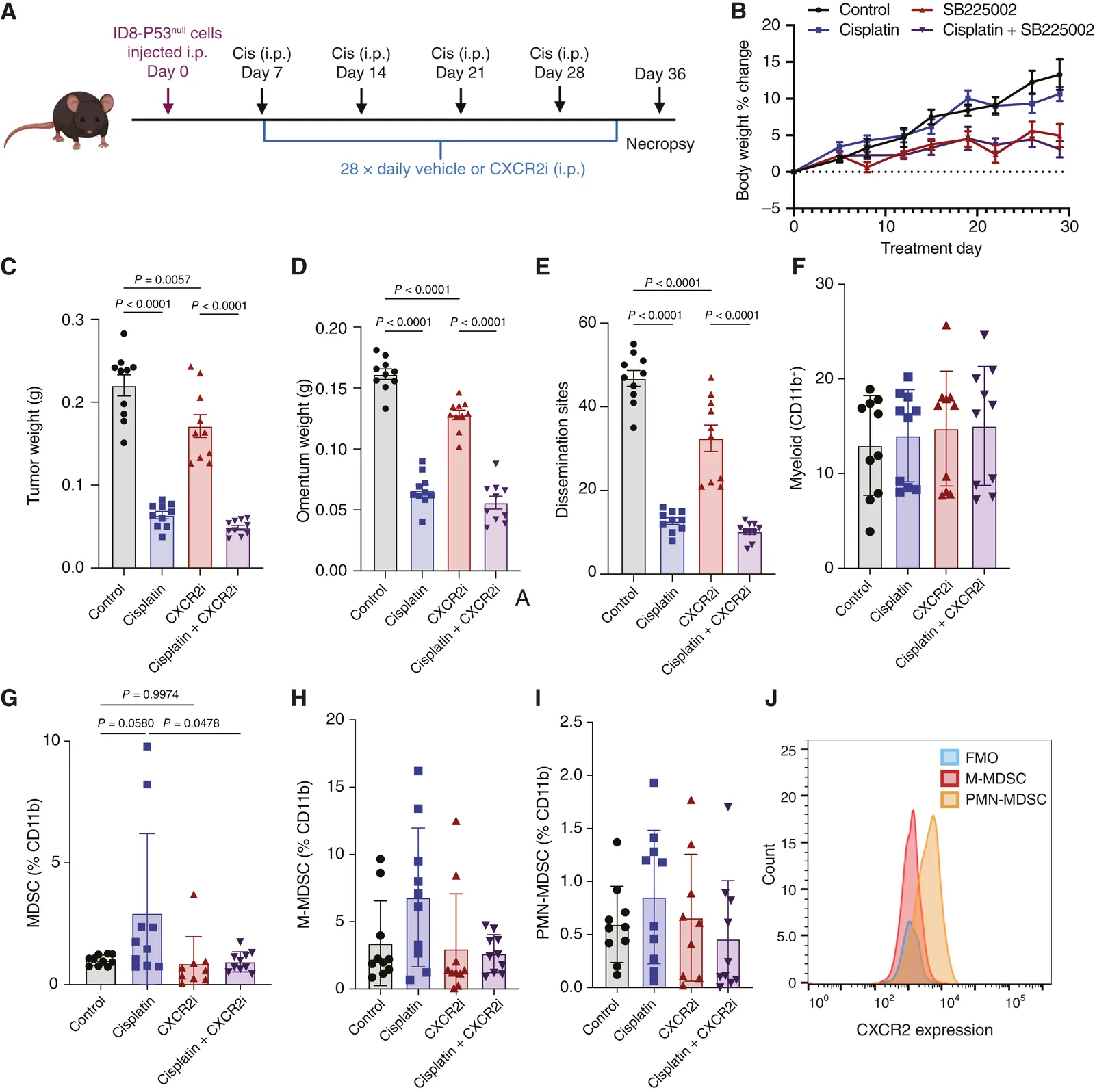
*In vivo* CXCR2i decreases tumor growth and dissemination and modulates the TIME. **A,** Treatment timeline of the ID8-p53^null^ murine model. **B,** Weight (g) of mice over treatment (**C**) total tumor weight Omentum weight (g) (**D**) omentum weight (g) (**E**) and tumor dissemination sites (number) of control and treatment tumors. **F,** Total myeloid cells (CD11b^+^) by treatment group. **G,** Total MDSCs as a percentage of CD11b^+^ cells by treatment group. **H,** M-MDSCs (CD11b^+^, Ly6G−, Ly6C^high^) as a percentage of CD11b^+^ populations by treatment groups. **I,** PMN-MDSCs (CD11b^+^, Ly6G^+^, Ly6C^low^) as a percentage of CD11b^+^ populations by treatment groups. **J,** CXCR2 expression on the cell surface of M-MDSCs and PMN-MDSCs by flow cytometry. Error bars, SD. Statistical test: (**B–H**) One-way ANOVA with Tukey multiple comparison test.

Given the role of CXCR2 in the invasion of immune cells into the TIME and its correlation with survival in human HGSC, we evaluated the role of CXCR2 in the ID8-p53^null^ murine model of HGSC, a well-established immune-intact HGSC murine model (20). We determined the effects of CXCR2i treatment on immune cell infiltration, tumor growth, and intraperitoneal dissemination. Female C57BL/6 tumor-bearing mice were treated with vehicle, cisplatin (1 mg/kg intraperitoneally, weekly), CXCR2i (4 mg/kg intraperitoneally, daily), or a combination of cisplatin and CXCR2i (Fig. 4A). The treatment was tolerated well in all mice, with a <10% weight loss in any group (Fig. 4B). Compared with control-treated mice, average tumor weights were decreased with single-agent cisplatin (mean tumor weight: 0.22 vs. 0.06 g for control vs. cisplatin, respectively; *P* < 0.001) and single-agent CXCR2i (mean tumor weight: 0.22 vs. 0.17 g for control vs. CXCR2i, respectively; *P* = 0.006); however, the combination of cisplatin and CXCR2i did not significantly change tumor weight compared with cisplatin alone (mean weight: 0.064 vs. 0.049 g for cisplatin vs. combination, respectively; *P* = 0.689; Fig. 4C). The changes in average total tumor weight are consistent with omentum weight, the primary site of initial tumor establishment (Fig. 4D). Similarly, intraperitoneal dissemination was decreased with single-agent cisplatin (mean number: 46.8 vs. 12.7 sites for control vs. cisplatin, respectively, *P* < 0.001) and single-agent CXCR2i (mean number: 46.8 vs. 32.5 sites for control vs. CXCR2i, respectively; *P* < 0.001); however, the combination of CXCR2i and cisplatin did not significantly affect dissemination compared with cisplatin alone (mean number: 12.7 vs. 10.2 sites for cisplatin vs. combination; *P* = 0.795; Fig. 4E). To determine if these effects were due to direct toxicity of the CXCR2i in the ID8-p53^null^ cells, a dose-response viability assay was performed *in vitro*, demonstrating no changes in cell viability at doses up to 1 μmol/L, a supraphysiologic dose (Supplementary Fig. S2B). To confirm these *in vivo* results, this experiment was performed in the HGS2 (PAX8-Cre; p53^−/−^; Pten^−/−^; Brca2^−/−^) cell line model of HGSC, which showed a similar trend (Supplementary Fig. S2C and S2D). Targeting CXCR2 in the ID8 model did not result in any overt toxicity measured by changes in body weight (Fig. 4B). These results demonstrate that targeting CXCR2 alone is tolerated and is sufficient to inhibit HGSC tumor progression in mouse models of HGSC.

To determine how CXCR2 inhibition affects immune cell infiltration, omental tumors were separately evaluated by flow cytometry and IHC. The omental tumors were digested and evaluated using two fluorescent panels to quantify the proportions of myeloid- and lymphoid-derived cells (Supplementary Table S1). For myeloid cells, we evaluated MDSCs, including M-MDSC (CD11b^+^Ly6C^+^Ly6G^−^) and PMN-MDSC (CD11b^+^Ly6C^lo^Ly6G+). For lymphoid-derived cells, we evaluated CD3^+^ T cells, CD8^+^ T cells (CD3^+^CD8^+^CD4^−^), CD4^+^ T cells (CD3^+^CD8^−^CD4^+^), and NK cells (NK1.1+). Although there was no global change in tumor-associated myeloid cells (Fig. 4F), there was a trend toward increased MDSCs in the cisplatin-treated mice (1% vs. 2.96% MDSC for control vs. cisplatin, respectively; *P* = 0.058), which was abrogated by the addition of CXCR2i to cisplatin (2.96% vs. 0.94% MDSC for cisplatin vs. combination, respectively; *P* = 0.048; Fig. 4G). However, there was no significant decrease in MDSC infiltration in control- versus CXCR2i-treated mice (1% vs. 0.86% MDSC for control vs. CXCR2i, respectively; *P* = 0.997; Fig. 4G). This trend, increased MDSCs with cisplatin inhibited by the addition of CXCR2i, was consistent across the M-MDSC and PMN-MDSC phenotypes individually although not statistically significant (Fig. 4H and I). Interestingly, a negative selection kit was used to isolate MDSCs from the bone marrow of tumor-bearing mice, and only PMN-MDSCs expressed CXCR2, suggesting a nondirect role of the CXCR2i on the M-MDSC population (Fig. 4J). No changes in tumor-associated T cells were observed (Supplementary Fig. S2E–S2G). As a measure of systemic changes, the spleens of the mice from each group were evaluated, and no differences existed in the immune cell populations between treatment groups (Supplementary Fig. S2H–S2J).

**Figure 5.**
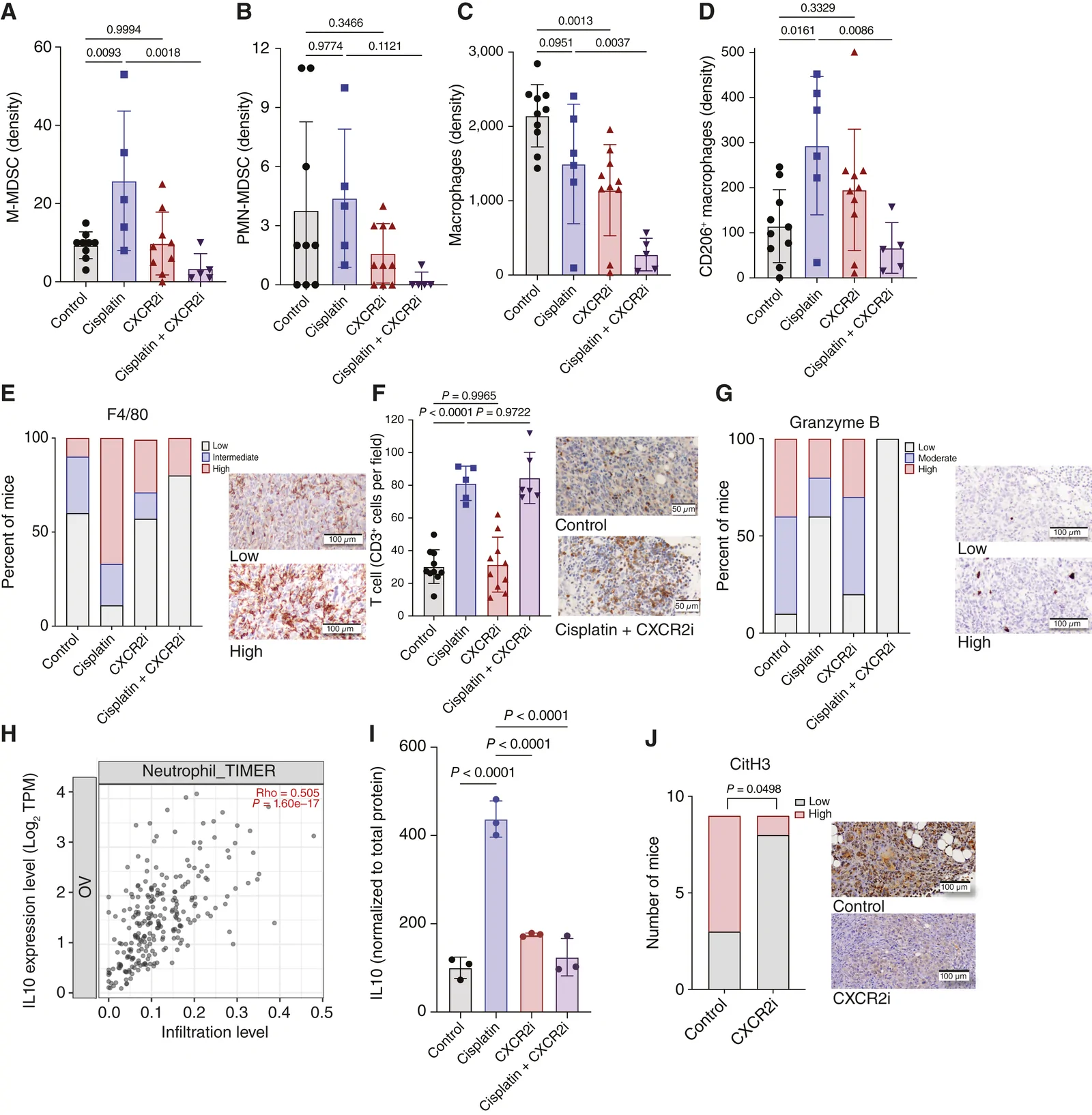
mIHC and conventional IHC demonstrate modulation of the TIME by CXCR2i in ID8-p53^null^ omental tumors. Cells were stained using a nine-color panel, and the density of cell types was quantified. Data are presented for (**A**) M-MDSCs (CD11b^+^/MHCII^−^/Ly6C^+^), (**B**) PMN-MDSCs (CD11b^+^/MHCII^−^/Ly6G^+^), (**C**) macrophages (F4/80^+^), and (**D**) CD206^+^ macrophages (F4/80^+^/CD206^+^; *n* = 10 control, *n* = 10 CXCR2i, *n* = 6 cisplatin, *n* = 5 combination). **E,** Pathologist review of F4/80 conventional IHC by treatment group, split into high, intermediate, and low expression. **F,** Quantification of the percentage of CD3^+^ T cells from conventional IHC by treatment group. **G,** Pathologist review of granzyme B by conventional IHC by treatment group, split into high, intermediate, and low expression. **H,***IL10* gene expression determined by RNA-seq in The Cancer Genome Atlas samples correlated with infiltration levels of neutrophils in tumors of patients with ovarian cancer. **I,** Blinded review of citH3 split into low and high expression. **J,** Production of IL10 measured by ELISA from whole digested tumors normalized to total protein. Error bars, SD. Statistical tests: (**A–D** and **F**) One-way ANOVA with Tukey multiple comparison test and (**E**, **G**, and **H**) Fisher exact test.

Although flow cytometry evaluates whole tissue to obtain a global understanding and quantification of immune cells, the requirement to dissociate tissue may cause potential loss of rare cells or cells that are more sensitive to digestion, as well as high background signals due to cell death during the digestion process. To address these technical limitations, we performed mIHC to characterize the myeloid cell compartment in the TIME. We designed a nine-parameter panel (Supplementary Table S4) to evaluate macrophage and MDSC infiltration in treated and untreated tumors. Similar to the results generated by flow cytometry, mIHC showed that the average density of M-MDSCs increased with cisplatin treatment (mean density: 9 vs. 25.8 for control vs. cisplatin, respectively; *P* = 0.009), and this cisplatin-mediated increase was inhibited by the addition of the CXCR2i (mean density: 25.8 vs. 3.4 for cisplatin vs. combination, respectively; *P* = 0.002; Fig. 5A). This pattern was also present in the PMN-MDSC population but did not meet statistical significance although there was almost complete depletion of PMN-MDSCs in the cisplatin + CXCR2i combination group (Fig. 5B). Unexpectedly, CXCR2i also decreased macrophage tumor infiltration compared with control (mean density: 2,143 vs. 1,142 cells/mm^2^ for control vs. cisplatin, respectively; *P* = 0.003), and the addition of CXCR2i to cisplatin decreased macrophage infiltration compared with single-agent cisplatin (mean density: 1,495 vs. 276 cells/mm^2^ for cisplatin vs. combination, respectively; *P* = 0.007; Fig. 5C). Furthermore, CD206 denoted the immunoinhibitory macrophage population. Compared with control, the CD206^+^ macrophages increased in the setting of cisplatin (mean density: 114.7 vs. 293.2 cells/mm^2^ for control vs. cisplatin, respectively; *P* = 0.016), and this cisplatin-mediated increase was again inhibited with the CXCR2i (mean density: 293.2 vs. 66.4 cells/mm^2^ for cisplatin vs. combination, respectively; *P* = 0.009; Fig. 5D), mirroring the prior MDSC results. Conventional IHC further validated our finding that macrophage infiltration was increased by cisplatin and that the cisplatin-mediated increase in macrophages was abrogated by CXCR2i (Fig. 5E). On conventional IHC, CD3^+^ T cells increased with the addition of cisplatin compared with control, with no change in T-cell infiltration with single-agent CXCR2i compared with control or the combination of CXCR2i with cisplatin compared with single-agent cisplatin (Fig. 5F). Interestingly, granzyme B, a marker of activated immune cells (i.e., involved in an antitumor response), was increased with the treatment of cisplatin compared with control but decreased with the addition of CXCR2i (Fig. 5G). These data from the *in vivo* model were consistent with the *in vitro* studies demonstrating the importance of CXCR2 for myeloid cell migration.

Although the *in vivo* data demonstrate a cisplatin-mediated migration of immunosuppressive myeloid cells, inhibited by CXCR2i, this does not demonstrate a functional role of these myeloid cells. One of the major cytokines released by immunoinhibitory macrophages, MDSCs, and immature neutrophils is IL10, a potent immunosuppressive cytokine (41–43). When detected in high levels in the ascites of patients with ovarian cancer, IL10 is correlated with increased disease progression, decreased time to recurrence, and decreased OS (44, 45). Furthermore, using TIMER V2, IL10 gene expression is correlated with neutrophil infiltration (Rho = 0.505, *P* < 0.0001; Fig. 5H). To assess IL10 production from tumors as a functional marker of the inhibitory immune cells, tumor establishment and treatment were performed as described (Fig. 4A). Tumors were then resected and digested into single cells and plated for 24 hours, at which point supernatants were collected from the cell cultures, and an ELISA was performed for IL10 concentration, normalized to total protein. Compared with control, average IL10 secretion increased with cisplatin treatment, and the cisplatin-mediated increase in IL10 was prevented by the addition of CXCR2i (Fig. 5I). These data are consistent with cisplatin leading to functional immunoinhibitory immune cells, which is reversed by the addition of CXCR2i.

Although CXCR2i as a single agent decreased the weight and distribution of ID8-p53^null^ tumors, it did not affect MDSC infiltration into the tumors, suggesting an alternative mechanism of action when used as a single agent. CXCR2 is a mediator of NETosis, a type of granulocyte (i.e., neutrophil and PMN-MDSC) programmed cell death that leads to the extrusion of DNA and proteins that form neutrophil extracellular traps (NET). In solid tumors, NETs promote tumor growth by inhibiting CD8^+^ T-cell interactions with the tumor cells, thereby protecting the cancerous cells from immune-mediated cytotoxicity (46). CXCR1 and CXCR2 are the main mediators of tumor-induced NETosis (46). We performed conventional IHC for citrullinated histone H3 (citH3), a marker of NETosis, on the tumors from the control- and CXCR2i-treated mice. Given the minimal tumor in the cisplatin and cisplatin + CXCR2i groups, citH3 analysis on these slides was not reliable. However, in tumors treated with CXCR2i alone, there was a significant decrease in tumor-associated NETosis compared with controls (Fig. 5J). This finding demonstrates that CXCR2 activation has multiple downstream mechanisms in granulocytes, including activation of chemotaxis as well as NETosis once the granulocytes reach the site of inflammation.

## Discussion

The TME plays an integral role in HGSC progression and response to treatment. Neutrophils, macrophages, and MDSCs are potent immune suppressors in the TME, causing increased tumor growth, spread, and resistance to treatment (5, 33, 47, 48). Modulating these immunosuppressive cell populations represents an important and clinically relevant approach to improving patient treatment response and survival by relieving immunosuppression to promote immune clearance of tumor cells. The findings presented here implicate the chemokine receptor CXCR2 as a new targetable node in the immunosuppressive TME of HGSC.

To evaluate how the infiltration of immunosuppressive cells affects treatment response and recurrence rates in human HGSC, we correlated the density of neutrophils and MDSCs with patient outcomes in a well-annotated TMA of human HGSC (24, 32). We found that increased neutrophil infiltration was correlated with decreased time to first recurrence, whereas MDSCs were not correlated with recurrence or survival. MDSCs by IHC have previously been correlated with survival; however, the methods used were less rigorous, using a single antibody to define tumor-associated MDSCs (9). Therefore, these data represent the most complex interrogation of MDSCs in the human HGSC TME to date by IHC. It is important to note the limitations of IHC, as cell surface markers can predict function but do not define the function of the cells. Furthermore, MDSCs, specifically PMN-MDSCs, defining surface markers have been extensively debated in the literature and are difficult to distinguish from neutrophils by surface markers (17). However, these data demonstrate the importance of neutrophils, the primary CXCR2-expressing cell, in disease progression, making CXCR2 a potential therapeutic target.

We also used publicly available RNA-seq data and determined that *CXCR2*, a major chemotactic receptor for neutrophil and MDSC migration, is correlated with decreased OS and increased intratumoral neutrophils, monocytes, and immunoinhibitory macrophages. These data suggest that CXCR2 plays a role in immune cell migration to tumors and that its expression has a negative impact on survival in patients with HGSC. Given these data, we utilized a mouse system to study the impact of the CXCR2 receptor on MDSC generation and migration, as well as on tumor growth and intraperitoneal metastasis.

*In vitro*, we generated bone marrow–derived primarily PMN-MDSCs/tumor-educated neutrophils, as well as a smaller population of M-MDSCs. We confirmed their identity by evaluating cell surface markers and ensured their immunosuppressive phenotype by performing functional assays. Inhibition of CXCR2 did not affect the phenotypic or cell surface marker profiles of the bone marrow–derived MDSCs; however, CXCR2i treatment did reduce the migratory and invasive characteristics of MDSCs. These results are consistent with the known function of CXCR2 (49). To further evaluate the effects of CXCR2i on bone marrow–derived MDSC function, we isolated MDSCs from the bone marrow of tumor-bearing mice, with or without 28 days of treatment with the CXCR2i, and then analyzed differences in protein expression and signaling. Some identified proteins of interest were consistent with expected findings; for example, the CXCR2i-treated groups showed downregulation of both MAPK signaling (i.e., phosphorylated p38) and ENO1, which is important for neutrophil migration and invasion at sites of inflammation (50–52). However, there were also changes in protein expression with CXCR2i in pathways not previously described in immune cells. Therefore, future studies may be performed to evaluate the role of these proteins in MDSCs and their function in developing novel targets to modify these cancer-promoting granulocytic cells.

To determine the effects of the CXCR2i *in vivo*, we evaluated the role of a CXCR2i in a murine HGSC model. We observed that as a single agent, CXCR2i reduced tumor burden and intraperitoneal spread of ID8-p53^null^ cells, but given the clearance of tumor by cisplatin alone, there was no additive benefit of the CXCR2i with cisplatin. The most intriguing changes in the TIME, measured by flow cytometry and IHC, were in the mice treated with cisplatin and with a combination of cisplatin and CXCR2i. Compared with control, cisplatin treatment increased CD8^+^ granzyme B^+^ T cells, consistent with activated, cytotoxic T cells (53), and concomitantly increased the infiltration of immune inhibitory cells including PMN-MDSCs/neutrophils, M-MDSCs, and CD206^+^ macrophages, resulting in an increase in IL10 secretion, a potent immunosuppressive cytokine. Therefore, although chemotherapy has the potential to activate a T-cell response against the tumor, it also causes an increase in immune inhibitory myeloid cells, which can lead to immune evasion and increased tumor progression (18). In the combination group, the addition of CXCR2i to cisplatin does not change the increase in CD8^+^ granzyme B^+^ T cells; however, it does impede the infiltration of the immunosuppressive myeloid cells and the increased IL10 secretion described above. The combination of CXCR2i with cisplatin allows for an increased T-cell response to HGSC while inhibiting the accompanying myeloid cell inhibitory response.

Single-agent CXCR2i in the ID8-P53^−/−^ model of HGSC did not change the intratumoral immune cell milieu but did decrease tumor size and intraperitoneal metastasis. Using an *in vitro* ID8 cell viability assay, we demonstrated that the effects were not on the cancer cells directly. Therefore, we evaluated alternative mechanisms for CXCR2i to affect the TME. CXCR2i can decrease NET formation by neutrophils (14, 46), and NETs are associated with increased tumor growth and progression (46). We evaluated citH3, a marker of NET formation, by IHC on control and CXCR2i-treated tumors, with decreased citH3 in tumors from mice treated with the CXCR2i. These data demonstrate a nonchemotactic, tumor-promoting effect of CXCR2 through granulocytic cells. Taken together, these data demonstrate that the presence of neutrophils and PMN-MDSCs has an immune inhibitory impact on the TME of HGSC and that by blocking CXCR2, this immune inhibition can be reversed.

This study offers a new direction for potential modification of the immunosuppressive TME in HGSC, which has been notoriously difficult to overcome. Prior studies in HGSC have shown that after treatment with standard-of-care chemotherapy, there is an increase in T-cell infiltration, which was interpreted as an increased antitumor, proinflammatory response. However, more contemporary literature is consistent with our findings, showing a concomitant infiltration of immunosuppressive immune cells (54, 55). Inhibiting the influx of these immunosuppressive cells has the potential to improve response to treatment, lead to a longer progression-free interval, and allow a robust T-cell response, which is associated with improved survival (56). CXCR1/2is have been well tolerated in clinical trials to date and serve as a novel immune modifier in the context of HGSC.

Although the concept of using CXCR1/2is in solid tumors is not in itself novel, using a CXCR1/2i in the treatment of HGSC and in conjunction with upfront standard-of-care chemotherapy is novel. There have been multiple trials of CXCR1/2i in both malignant and nonmalignant contexts, demonstrating their safety. However, most trials evaluating CXCR1/2i in solid tumors have been in early-phase trials in a pretreated (often heavily pretreated) population (Table 1). We would assert that these trials failed to demonstrate a survival benefit because CXCR1/2is were being evaluated in tumors in which the TME has already been remodeled by chemotherapy. In contrast, we propose a novel paradigm in which a CXCR2i is used in the upfront treatment of HGSC in addition to standard-of-care chemotherapy to increase the cytotoxic T-cell population while preventing the chemotherapy-induced influx of tumor-promoting, immune inhibitory cells. Current immunotherapy approaches that primarily target T-cell immune checkpoints have been disappointing in HGSC, and modifying the myeloid population is a provocative new approach.

**Table 1.** Published clinical trials using CXCR1/2is in oncology.

Trial/year	Cancer type	Phase	Treatment	Number of patients	Lines of prior treatment, *n* (%)	Endpoints	Outcomes	AE (any grade)	AE G3 or higher
Schott and colleagues 2017 PMID: 28539464	HER2-negative metastatic breast cancer	Ib	Reparixin + paclitaxel	30	Prior CT: No, 12 (40) Yes, 18 (66) Prior CT Regimens: 0: 14 (46) 1: 9 (30) ≥2: 7 (23.3)	Response rate	ORR, 33% CR, 4%	No DLT	2.7%
Goldstein and colleagues 2020 PMID: 31924241	Operable HER2-negative breast cancer		Reparixin 21 days preoperatively	20	N/A	Effect on CSCs in primary tumor	Decrease in CSC > 20%	75%	5%
Goldstein and colleagues 2021 PMID: 34476645	Metastatic triple-negative breast cancer	II	Reparixin + paclitaxel (combo) vs. paclitaxel (Taxol)	123	Prior CT: 101 (82) Prior taxane: 85 (69)	PFS	Not met	Combo: 98% Taxol: 95%	Combo: 21% Taxol: 20%
Guo and colleagues 2023 PMID: 37844613	Castration-resistant prostate cancer	I/II	AZD5069 + enzalutamide	23	Prior radiation: 5 (21.7) Median lines of systemic therapy: 4	Dose finding	PR, 27% AZD5069 discontinued due to study termination		
Armstrong and colleagues 2024 PMID: 38324085	Solid tumors Advanced pancreatic MSS CRC Non–small cell lung cancer	II	Navarixin (30 or 100 mg) + pembrolizumab	105	Prior CT: Regimens: 1: 10 (10) 2: 27 (26) 3: 27 (26) 4: 25 (24) ≥5: 14 (13) Missing: 1	ORR DLT AE	ORR prostate 30 and 100 mg: 5% MSS CRC: 30 mg 2.5%	30 mg: 73% 100 mg: 61%	30 mg: 25% 100 mg: 22%

Abbreviations: AE, adverse events; CR, complete response; CRC, colorectal cancer; CSC, cancer stem cells; CT, chemotherapy; DLT, dose-limiting toxicity; MSS, microsatellite stable; ORR, objective response rate; PR, partial response.

## Supplementary Material

Figure S1 CXCR1, CXCR2 and MDSC correlation with survival and/or prognostic indicators.

Figue S2 CXCR2i affects the myeloid compartment of murine models of HGSC without effect on the systemic immune system

Table S1 Flow Cytometry Reagents

Table S2 Conventional mouse immunohistochemistry antibodies

Table S3 Human Multiplexed Immunohistochemistry Antibody Panel

Table S4 Mouse multiplexed immunohistochemistry antibody panel

## Data Availability

All the data generated in this study are presented in the article or supplemental information. Raw data are available from the corresponding author upon request.
