# Supplementary material for: Inhibiting CXCR2 Remodels the Tumor Microenvironment and Blunts Tumor Progression and Dissemination of High-Grade Serous Carcinoma
Source: Cancer Res Commun. 2026 Sep 24;6(9):2256–70. doi: 10.1158/2767-9764.CRC-26-0408 (PMC13602021; doi:10.1158/2767-9764.CRC-26-0408)
Supplement: Supplementary file 1 [file crc-26-0408_figue_s2_suppsf2.docx]

**Figure S2. CXCR2i affects the myeloid compartment of murine models of HGSC without effect on the systemic immune system.** **A)** *In vitro* MTT assay of ID8p53null cells treated with increasing concentrations of CXCR2i. **B)** *In vitro* MTT assay of MDSCs treated with increasing concentrations of CXCR2i **C)** The HGS2 model of HGSC was treated with control, cisplatin, CXCR2i and combination of cisplatin with CXCR2i and tumor weight and **D)** dissemination were evaluated. **E)** Total CD3+ T cells, **F)** CD4+ T cells and **G)** CD8+ T cells in the omental tumors of the ID8-p53null model were evaluated by flow cytometry. **H-J)** Flow cytometry analysis of spleen from ID8-p53null tumor bearing mice as an assessment of systemic immune system changes. **H)** T cell subsets **I)** NK cells **J)** Myeloid cells/MDSCs.
