# Supplementary material for: Inhibiting CXCR2 Remodels the Tumor Microenvironment and Blunts Tumor Progression and Dissemination of High-Grade Serous Carcinoma
Source: Cancer Res Commun. 2026 Sep 24;6(9):2256–70. doi: 10.1158/2767-9764.CRC-26-0408 (PMC13602021; doi:10.1158/2767-9764.CRC-26-0408)
Supplement: Supplementary file 2 [file crc-26-0408_figure_s1_suppsf1.docx]

**Figure S1** ***CXCR1*, *CXCR2* and** **MDSC correlation with survival and/or prognostic indicators**. Probability of overall survival versus time (in months) of HGSC patients using the TCGA cohort via KMPlotter stratified by **A)** *CXCR2*^high^ or *CXCR2*^low^ and **B)** *CXCR1*^high^ or *CXCR1*^low^. Multispectral IHC was performed on the HGSC TMA using markers to immunophenotype myeloid subsets and correlated with survival and prognostic indicators of survival **C)** MDSC correlated with PFS **D)** CD33^+^ cells correlated with OS **E)** Neutrophils correlated with PFS **F)** MDSCs correlated with optimal vs suboptimal debulking **G)** MDSCs correlated with neoadjuvant chemotherapy (NACT) vs upfront debulking surgery followed by adjuvant chemotherapy **H)** MDSCs correlation with germline BRCA mutation. Error bars, SD.
