# Supplementary material for: Inhibiting CXCR2 Remodels the Tumor Microenvironment and Blunts Tumor Progression and Dissemination of High-Grade Serous Carcinoma
Source: Cancer Res Commun. 2026 Sep 24;6(9):2256–70. doi: 10.1158/2767-9764.CRC-26-0408 (PMC13602021; doi:10.1158/2767-9764.CRC-26-0408)
Supplement: Supplementary file 3 [file crc-26-0408_table_s1_suppst1.docx]

| Antibody/ Reagent | Fluorophore | | Host/  isotype | | Clone | Supplier | Catalog # | RRID |
| --- | --- | --- | --- | --- | --- | --- | --- | --- |
| CD11b | APC-Cy7 | Rat IgG2a, k | | M1/70 | | Biolegend | 101226 | AB_830641 |
| CD3e | PE | Armenian Hamster | | 145-2C11 | | Biolegend | 100308 | AB_312672 |
| CD4 | PE-Cy7 | Rat IgG2a, k | | RM4-5 | | Biolegend | 100528 | AB_312729 |
| CD8a | APC | Rat IgG2a, k | | 53-6.7 | | Biolegend | 100712 | AB_312750 |
| CXCR2 | Cy5.5 | Rat IgG2a, k | | SA044G4 | | Biolegend | 149308 | AB_2565695 |
| Ly-6G | PE | Rat IgG2a, k | | 1A8 | | Biolegend | 164503; 127608 | AB_2904311  AB_1186099 |
| Ly-6C | FITC | Rat IgG2a, k | | HK1.4 | | Biolegend | 128006 | AB_1186134 |
| NK-1.1 | Alexa Fluor 700 | Mouse IgG2a | | PK136 | | Biolegend | 108730 | AB_2074426 |
| NOS2 (iNOS) | PE | Rat IgG2a, k | | W16030C | | Biolegend | 696805 | AB_2876745 |
| Fixable Vaibility stain 660 | APC | - | | - | | BD Biosciences | 564405 | AB_2869571 |
| FC blocker | - | - | | - | | Biolegend | 422301 | AB_2818986 |
| ZombieRed | - | - | | - | | Biolegend | 423109 | N/A |
| Zombie violet | - | - | | - | | Biolegend | 423114 | N/A |

**Table S1: Flow Cytometry Reagents**
