# Supplementary material for: Inhibiting CXCR2 Remodels the Tumor Microenvironment and Blunts Tumor Progression and Dissemination of High-Grade Serous Carcinoma
Source: Cancer Res Commun. 2026 Sep 24;6(9):2256–70. doi: 10.1158/2767-9764.CRC-26-0408 (PMC13602021; doi:10.1158/2767-9764.CRC-26-0408)
Supplement: Supplementary file 4 [file crc-26-0408_table_s2_suppst2.docx]

| Antibody | Host/ Isotype | Clone | Supplier | Catalog # | RRID |
| --- | --- | --- | --- | --- | --- |
| F4-80 | Rabbit | D2S9R | CST | 70076 | AB_2799771 |
| Granzyme B | Rabbit | EPR22645-206 | Abcam | ab255598 | AB_2860567 |
| CD3 | Rabbit | Polyclonal | Dako | A452 | AB_233567 |
| CitH3 | Rabbit | Polyclonal | Abcam | ab5103 | AB_304752 |

**TABLE S2 Conventional mouse immunohistochemistry antibodies**
