# Supplementary material for: Inhibiting CXCR2 Remodels the Tumor Microenvironment and Blunts Tumor Progression and Dissemination of High-Grade Serous Carcinoma
Source: Cancer Res Commun. 2026 Sep 24;6(9):2256–70. doi: 10.1158/2767-9764.CRC-26-0408 (PMC13602021; doi:10.1158/2767-9764.CRC-26-0408)
Supplement: Supplementary file 5 [file crc-26-0408_table_s3_suppst3.docx]

| Antibody | Fluorophore | Host/ Isotype | Clone | Supplier | Catalog # | RRID |
| --- | --- | --- | --- | --- | --- | --- |
| CD11b | Opal 570 | Rabbit, IgG | D6X1N | CST | 49420S | AB_2799357 |
| CD14 | Opal 690 | Rabbit, IgG | SP192 | Abcam | ab183322 | AB_2909463 |
| CD15 | Opal 520 | Mouse, IgM | MMA | Abcam | ab17080 | AB_443635 |
| CD33 | Opal 650 | Mouse, IgG2b | PWS44 | Leica | PA0555 | AB_10555285 |
| CD68 | Opal 480 | Mouse | KP1 | Dako | M0814 | AB_2314148 |
| HLA-DR | Opal 780 | Mouse, IgG1 | TAL 1B5 | Abcam | ab20181 | AB_445401 |
| CD66b | Opal 540 | Mouse, IgM k | G10F5 | Novus | NBP2-80664 | AB_3096017 |
| CK | Opal 620 | Mouse, IgM k | AE1/AE3 | Dako | M3515 | AB_2132885 |
| DAPI |  |  |  |  |  |  |

**TABLE S3: Human Multiplexed Immunohistochemistry Antibody Panel**
