# Supplementary material for: Inhibiting CXCR2 Remodels the Tumor Microenvironment and Blunts Tumor Progression and Dissemination of High-Grade Serous Carcinoma
Source: Cancer Res Commun. 2026 Sep 24;6(9):2256–70. doi: 10.1158/2767-9764.CRC-26-0408 (PMC13602021; doi:10.1158/2767-9764.CRC-26-0408)
Supplement: Supplementary file 6 [file crc-26-0408_table_s4_suppst4.docx]

| Antibody | Fluorophore | Host/ Isotype | Clone | Supplier | Catalog # | RRID |
| --- | --- | --- | --- | --- | --- | --- |
| CD11b | Opal 520 | Rabbit, IgG | Polyclonal | Novus | NB110-89474 | AB_1216361 |
| Ly6C | Opal 570 | Rat, IgG2a | ER-MP20 | BioRad | MCA2389T | AB_1102741 |
| Ly6G | Opal 690 | Rabbit, IgG | E6Z1T | CST | 87048S | AB_2909808 |
| F4/80 | Opal 780 | Rabbit, IgG | D4C8V | CST | 30325S | AB_2798990 |
| CD206 | Opal 480 | Rabbit, IgG | E6T5J | CST | 24595S | AB_2892682 |
| MHCII | Opal 540 | Rat IgG2b, k | M5/114.15.2 | BioLegend | 107601 | AB_313316 |
| CD11c | Opal 620 | Rabbit, IgG | D1V9Y | CST | 97585S | AB_2800282 |
| WT1 | Opal 650 | Mouse, IgG1 k | 6FH2 | Novus | NB110-60011B | AB_1849479 |
| DAPI |  |  |  |  |  |  |

**TABLE S4: Mouse multiplexed immunohistochemistry antibody panel**
